# Emerging therapeutic role of gut microbial extracellular vesicles in neurological disorders

**DOI:** 10.3389/fnins.2023.1241418

**Published:** 2023-08-09

**Authors:** Bowen Sun, Harshal Sawant, Alip Borthakur, Ji Chen Bihl

**Affiliations:** ^1^Departments of Biomedical Sciences, Joan C. Edwards School of Medicine, Marshall University, Huntington, WV, United States; ^2^Department of Neurosurgery, The First Affiliated Hospital, Harbin Medical University, Harbin, Heilongjiang, China; ^3^Departments of Clinical and Translational Sciences, Joan C. Edwards School of Medicine, Marshall University, Huntington, WV, United States

**Keywords:** gut-brain axis, gut bacteria, microbiota, extracellular vesicle, neurological disorder

## Abstract

Extracellular vesicles (EVs) serve as cell-to-cell and inter-organ communicators by conveying proteins and nucleic acids with regulatory functions. Emerging evidence shows that gut microbial-released EVs play a pivotal role in the gut-brain axis, bidirectional communication, and crosstalk between the gut and the brain. Increasing pre-clinical and clinical evidence suggests that gut bacteria-released EVs are capable of eliciting distinct signaling to the brain with the ability to cross the blood–brain barrier, exerting regulatory function on brain cells such as neurons, astrocytes, and microglia, via their abundant and diversified protein and nucleic acid cargo. Conversely, EVs derived from certain species of bacteria, particularly from gut commensals with probiotic properties, have recently been shown to confer distinct therapeutic effects on various neurological disorders. Thus, gut bacterial EVs may be both a cause of and therapy for neuropathological complications. This review marshals the basic, clinical, and translational studies that significantly contributed to our up-to-date knowledge of the therapeutic potential of gut microbial-derived EVs in treating neurological disorders, including strokes, Alzheimer’s and Parkinson’s disease, and dementia. The review also discusses the newer insights in recent studies focused on developing superior therapeutic microbial EVs via genetic manipulation and/or dietary intervention.

## 1. Introduction

The bidirectional communication and crosstalk between the gut and the brain, termed the gut-brain axis, has been well recognized. Mounting pre-clinical data and emerging clinical evidence suggest that gut microbiota plays a pivotal role in this bidirectional communication network, leading to the more recent concept of the gut-brain-microbiota axis which has a tremendous impact on the physiological and pathophysiological processes of both the organs (Heiss and Olofsson, 2019; Osadchiy et al., 2019; Margolis et al., 2021). In this regard, extracellular vesicles (EVs) produced by gut bacteria have been implicated as a potential critical regulator of interkingdom and interorgan communication, playing a key role in normal physiology and the pathophysiology of the brain and neurological functions (Pirolli et al., 2021). Gut microbiota-derived EVs (GMEVs) are capable of eliciting distinct signaling to the brain, possibly directly via crossing the blood–brain barrier (BBB), and exerting differential effects on brain cells such as neurons, astrocytes, and microglia, via their abundant and diversified protein and small ribonucleic acid (RNA) cargo (Srivastava and Kim, 2022). GMEVs have been shown to regulate brain gene expression and induce pathology at most stages of neuroinflammation and neurodegeneration, thus playing a causative role in various diseases such as stroke, Alzheimer’s disease (AD) and Parkinson’s disease (PD), and dementia (Lee et al., 2020; Yang et al., 2022). Conversely, EVs derived from certain species of bacteria, particularly gut commensals with probiotic properties, have recently been shown to confer distinct therapeutic effects on various neurological disorders (Park and Tsunoda, 2022). Thus, GMEVs may be a cause of as well as therapy for neuropathological complications. This review compiles the fundamental, clinical, and translational studies that have made substantial contributions to our up-to-date knowledge of the therapeutic potential of GMEVs in treating neurological disorders. It also provides information regarding the underlying mechanisms of beneficial effects and the applicability of circulating GMEVs as biomarkers of various neurological disease states. This review additionally discusses the latest findings in recent studies focused on the advancement of enhanced therapeutic bacterial EVs via genetic manipulation and/or dietary intervention.

## 2. Role of the microbiota in gut-brain axis: microbiota-gut-brain axis

The “gut–microbiota–brain axis” refers to the network of connections involving multiple biological systems that allow bidirectional communication between gut bacteria and the brain, and is crucial in maintaining homeostasis of the gastrointestinal, central nervous, and microbial systems of animals (Morais et al., 2021). These processes may affect human health, as certain animal behaviors appear to correlate with the composition of gut bacteria, and disruptions in microbial communities have been implicated in several neurological disorders (Foster et al., 2017; Cryan et al., 2020). Emerging evidence suggests that the gut microbiota is intimately involved in the pathology of a wide range of neurological disorders and gut microbiota dysbiosis is a risk factor. Although structure and space may limit the contact between the microbiome and the intestinal epithelium, the substances secreted by the microbiome can pass through the limitations of space and serve as important factors in the function of the gut-brain axis. Various microbial products, including metabolites and secreted proteins, can cross the mucin layer to reach the host cells on the mucosal surface of the intestine. More recently, EVs have emerged as a novel communicator of the gut-microbiota-brain axis.

## 3. Gut microbiota-released extracellular vesicles

The gut microbiota can produce EVs in both physiological and pathological situations. Mounting evidence suggests that interkingdom crosstalk is principally mediated by EVs released by gut microbiota or by host intestinal cells (Diaz-Garrido et al., 2021).

### 3.1. Origin and type GMEVs

Traditionally, bacteria are categorized into two classes, according to their outer membrane nature: Gram-negative (G−) and Gram-positive (G+) bacteria (Sultan et al., 2021). The structure of G− bacteria is characterized by a double plasma membrane separated by the surrounding periplasm. The main types of EVs that are secreted come from the outer membrane and are called “outer membrane vesicles” (OMVs; Schwechheimer and Kuehn, 2015; Jan, 2017). OMVs are spherical particles consisting of the outer lobe of lipopolysaccharide (LPS) and the inner lobe of phospholipid. They originate from outer membrane bubbles, so they are enriched in the outer membrane and periplasmic biomolecules (Kulkarni and Jagannadham, 2014; Schwechheimer and Kuehn, 2015; Volgers et al., 2018). Some G− bacteria produce another type of EVs that contains fragments from the cytoplasm and periplasmic membrane and are rich in adenosine triphosphates and deoxyribonucleic acid (DNA), called inner and OMVs (Pérez-Cruz et al., 2015). In addition, recent studies have described a new type of G− bacteria-derived vesicles called “explosive outer-membrane vesicles” by the biogenesis mechanism (Toyofuku et al., 2019). The membrane of G+ bacteria has different properties from that of G− bacteria and the special thick layer of its peptidoglycan is thought to be an obstacle in the production of EVs. However, Lee EY et al. discovered EVs from *Staphylococcus aureus* and found that the mechanism of EVs released by G+ bacteria was also different from that of G− bacteria. EVs were produced through dilation, protease cleavage, or protein channels being pushed through thick membranes (Lee et al., 2009; Vallejo et al., 2012). Studies on a variety of G+ bacteria, such as *Staphylococcus aureus*, *Bacillus anthracis*, *Listeria monocytogenes*, *Clostridium perfringens*, *Bacillus subtilis*, have confirmed that the EVs derived from G+ bacteria are also spherical membrane particles with a diameter of 20–100 nm (Lee et al., 2009; Brown et al., 2015; Kim et al., 2015). Because of the lack of an outer membrane, G+ bacteria secreted EVs are called “cytoplasmic membrane vesicles” (CMVs; Toyofuku et al., 2019). EVs from G+ bacteria, despite lacking LPS and periplasmic components, carry similar types of cargo molecules to G− bacteria EVs, including peptidoglycans, lipids, proteins, and nucleic acids (Brown et al., 2015).

### 3.2. Diversity and complexity of molecular species in GMEVs cargo

**Figure 1:**
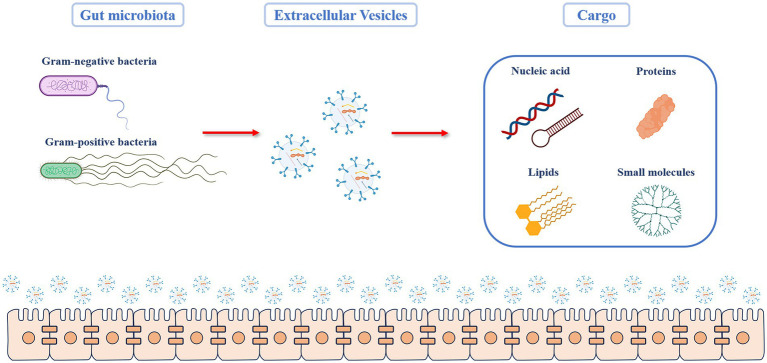
Cargo components of GMEVs. The Gut microbiota includes gram-positive and gram-negative bacteria, both of which can produce extracellular vesicles. The molecular components carried by the GMEV cargo can be broadly divided into four categories. (1) Proteins: they include structural proteins, porins, ion channel proteins, transporters, enzymes, and stress-related proteins. (2) Lipids: they are mainly the components of the bacterial envelope, such as lipids involved in membrane binding, such as glycerol, phospholipid, and phosphatidylglycerol (3) Nucleic acids: this includes deoxyribonucleic acid or ribonucleic acid that can mediate targeted gene transfer to other cells. (4) Other small molecules, including bacterial metabolites. GMEVs: gut microbiota-released extracellular vesicles.

EVs are known to mediate communication between cells, and the effects of EVs depend on the cargo they carry. The composition of the cargo varies with respect to the type of bacteria, their growth, environmental conditions, and biogenetic mechanisms. GMEVs cargo transported includes proteins, lipids, nucleic acids, and small molecules, and each plays a different role (Figure 1; Bonnington and Kuehn, 2014).

#### 3.2.1. Proteins

The proteins carried by GMEVs depend on the bacteria’s classification and specific characteristics. Currently, proteins related to many biological processes have been identified, which can be divided into structural proteins, pore proteins, ion channels, transport proteins, enzymes, and proteins related to stress response according to their functions (Lee et al., 2008). EVs released by G-bacteria are enriched in outer membrane proteins such as out membrane protein (Omp)-A, Omp-C, and Omp-F and periplasmic proteins such as AcrA and alkaline phosphatase (Choi et al., 2011; Altindis et al., 2014; Jang et al., 2014). The vesicles released by G− bacteria and symbiotic strains contain proteins that contribute to intestinal colonization, competition, and bacterial survival, as well as regulate host immune and defense responses, and some proteins are strain-specific (Lee et al., 2007; Aguilera et al., 2014; Elhenawy et al., 2014; Zakharzhevskaya et al., 2017; Hong et al., 2019). Proteins involved in vesicle biogenesis or regulating vesicles’ size, yield, and load were found in enterococcus serotype typhoid OMVs (Nevermann et al., 2019). The probiotics *Escherichia coli* Nissle 1917 (EcN) carry the protein encoded explicitly by the strain, most of which are adhesins and adaptive factors, which help the bacteria grow and survive in the harmful intestinal environment (Aguilera et al., 2014; Hong et al., 2019). In addition to intracellular activity, these proteins also have functions related to host interaction and tissue colonization (Jeffery, 2018).

The protein analysis of some G+ bacteria has identified membrane and cytoplasmic proteins related to several biological processes, mainly including GMEVs from pathogens, which are full of pathogenic factors and toxins, and have pathogenicity (Lee et al., 2009; Rivera et al., 2010; Olaya-Abril et al., 2014; Jeon et al., 2016; Resch et al., 2016; Surve et al., 2016; Dean et al., 2019, 2020; West et al., 2020). CMVs transporter isolated from several G+ probiotics can be responsible for their beneficial role in immune regulation and host interaction (Lee et al., 2016; Behzadi et al., 2017; Domingues and Nielsen, 2017; Li et al., 2017).

#### 3.2.2. Lipids

The lipid is the main component of the bacterial envelope. It is generally believed that outer membrane phospholipids exist in the OMVs of G− bacteria. There is evidence that OMVs contain lipids not found in the bacterial outer membrane (Tashiro et al., 2011; Chowdhury and Jagannadham, 2013). The study of EcN showed that OMVs include lipids involved in membrane bindings, such as glycerol, phospholipids, phosphatidylglycerol, phosphatidylethanolamine, and cardiolipin. The structure of fatty acids can affect the fluidity and rigidity of lipid membranes (Barák and Muchová, 2013). LPS is a component of G− bacteria OMVs. *Porphyromonas gingivalis* OMVs carry LPS molecules with long sugar chains and diacyl lipid A (Haurat et al., 2011).

Similarly, diacyl lipid A accumulates in *Salmonella* OMVs (Elhenawy et al., 2014). In G+ bacteria, the lipid composition of CMVs varies with the type of bacteria. This may be related to the adaptation and survival of bacteria in the ecological environment (Coelho et al., 2019). In *Bacillus anthracis* and *Streptococcus pneumoniae*, vesicles are rich in C12–C16 chain saturated fatty acids (Olaya-Abril et al., 2014). Lipids containing unsaturated fatty acids are more abundant in vesicles derived from *Listeria monocytogenes* than in bacterial cells (Coelho et al., 2019).

#### 3.2.3. Nucleic acid

GMEVs derived from G– and G+ bacteria are loaded with DNA and RNA. GMEVs can transfer DNA to other bacterial cells, thus mediating targeted gene transfer (Domingues and Nielsen, 2017). The nucleic acids contained in GMEVs interacts with specific intracellular receptors in host cells, thus triggering the host immune response (Gilmore et al., 2021). GMEVs contain most lumen DNA genes corresponding to specific functions related to antibiotic resistance, virulence, and stress response (Bitto and Kaparakis-Liaskos, 2017). In *Clostridium perfringens*, GMEVs can encode bacterial toxins, such as α-Toxin and aerolysin O (pfoA; Jiang et al., 2014). RNA in GMEVs can also regulate the host immune system and other cellular processes. Small non-coding RNA contained in GMEVs mainly regulates post-transcriptional gene expression, while RNA vectors can induce phenotypic changes in target cells (Lee, 2019). In some pathogenic bacteria, small RNA secreted by GMEVs can mediate host immune response disorder (Koeppen et al., 2016; Choi et al., 2017). Many small non-coding RNA sequences transported in GMEVs of the probiotic *E. coli* Nissle (EcN) are consistent with human genome regions involved in the epigenetic mechanism or gene expression regulation related to the cell-specific transcriptional control (Celluzzi and Masotti, 2016).

#### 3.2.4. Small molecules

GMEVs are also loaded with metabolites and effectors that can regulate the function of target cells. These small molecules are selectively packaged according to the producer strains (Zakharzhevskaya et al., 2017). The effector molecules packaged in GMEVs can help bacteria survive in a specific niche. GMEVs of fragilis can be loaded with the antibacterial peptide bionic self-assembling peptide (BSAP), thus inhibiting the activity of other *Bacteroides* in the human intestine (Chatzidaki-Livanis et al., 2014). GMEVs can also mediate hydrophobic quorum-sensing molecules to promote communication in bacterial communities and control toxicity or biofilm formation (Brameyer et al., 2018).

### 3.3. The function of GMEVs: good or bad?

GMEVs involve many biological functions, varying depending on their specific cargo contents. The diversity of cargo molecules endows GMEVs to play a crucial role in bacteria-bacteria and bacteria-host interactions (Schwechheimer and Kuehn, 2015; Jan, 2017; Caruana and Walper, 2020). In terms of the interaction between bacteria and bacteria, GMEVs can help host bacteria maintain their niche by competing with or killing other bacteria. It can serve as a microbial defense mechanism to protect against harmful substances, including bacteriophages, antibiotics, reactive oxygen species, and antimicrobial peptides. It can also be used as a bait for pollutants or antibiotics against bacterial membrane to prevent harmful effects (Giordano et al., 2020). GMEVs also release antibiotic-resistant enzymes, which are beneficial to the producer strains and other susceptible bacteria in the microbial community, such as *Staphylococcus aureus* and *Bacteroides* GMEVs β-galactosidase enzyme (Lee et al., 2013; Stentz et al., 2015). GMEVs also contain hydrolytic enzymes, which catalyze the degradation of proteins and complex polysaccharides existing in the environment and help the microbial community obtain nutrients (Elhenawy et al., 2014). In addition, GMEVs nucleic acid may possess β-lactam gene, which codes for antibiotic resistance gene, and transfers at intraspecific and interspecific levels (Chatterjee et al., 2017).

In addition to the beneficial effects on bacterial communities, some GMEVs can regulate their predatory function. The gut microbiota produces EVs that carry messages of antibiotic resistance to surrounding bacteria (Schaar et al., 2014; Stentz et al., 2015). They can also carry degrading enzymes, such as murein hydrolase, peptidoglycan hydrolase, or endopeptidase, thus killing competitive beneficial bacteria (Jan, 2017). For example, GMEVs of some *Micrococcus* contain factors with antibacterial activity and a variety of hydrolases that lead to the cell lysis of target bacteria, thus having a predatory activity (Evans et al., 2012; Marshall and Whitworth, 2019).

## 4. Role of GMEVs in microbiota-host communication: inter-kingdom signaling

GMEVs interact with the host primarily through full cooperation in the host cytoplasm, activation of host receptors, and delivery of its bacterial content (O’Donoghue and Krachler, 2016). GMEVs can penetrate the eukaryotic cell membrane and intestinal cell wall (Stentz et al., 2015). It can be swallowed by immune cells in the lamina propria and has been detected in blood and urine. DNA of bacterial origin can be detected in the serum and plasma of healthy subjects (Shen et al., 2012; Gosiewski et al., 2017; Park et al., 2017). This shows that GMEVs can penetrate the intestinal epithelium and vascular endothelium and reach the distant part of the host. It is currently believed that GMEVs can pass through the intestinal wall through paracellular and transcellular pathways (Stentz et al., 2015). GMEVs can change the composition of tight junctions. Through tight junctions, the parent pathogen invades the intestinal epithelium. The vesicles of *Campylobacter jejuni* break down the junction proteins E-cadherin and occludin, causing the invasion of *Campylobacter jejuni* (Elmi et al., 2016). The vesicles of symbiotic bacteria can also increase the expression of tight junction proteins and limit the transport beside cells (Günzel and Yu, 2013). The probiotic EcN strain can produce OMVs and regulate the expression of tight junction proteins zonula occludens (ZO)-1 and ZO-2 in intestinal epithelial cells (Alvarez et al., 2016). In addition, GMEVs can also enter host cells through endocytosis, including reticulin-mediated, actin-dependent, caveolin-mediated, and reticulin-independent endocytosis (Zingl et al., 2021).

## 5. Role of GMEVs in gut-brain axis: inter-organ signaling

**Figure 2:**
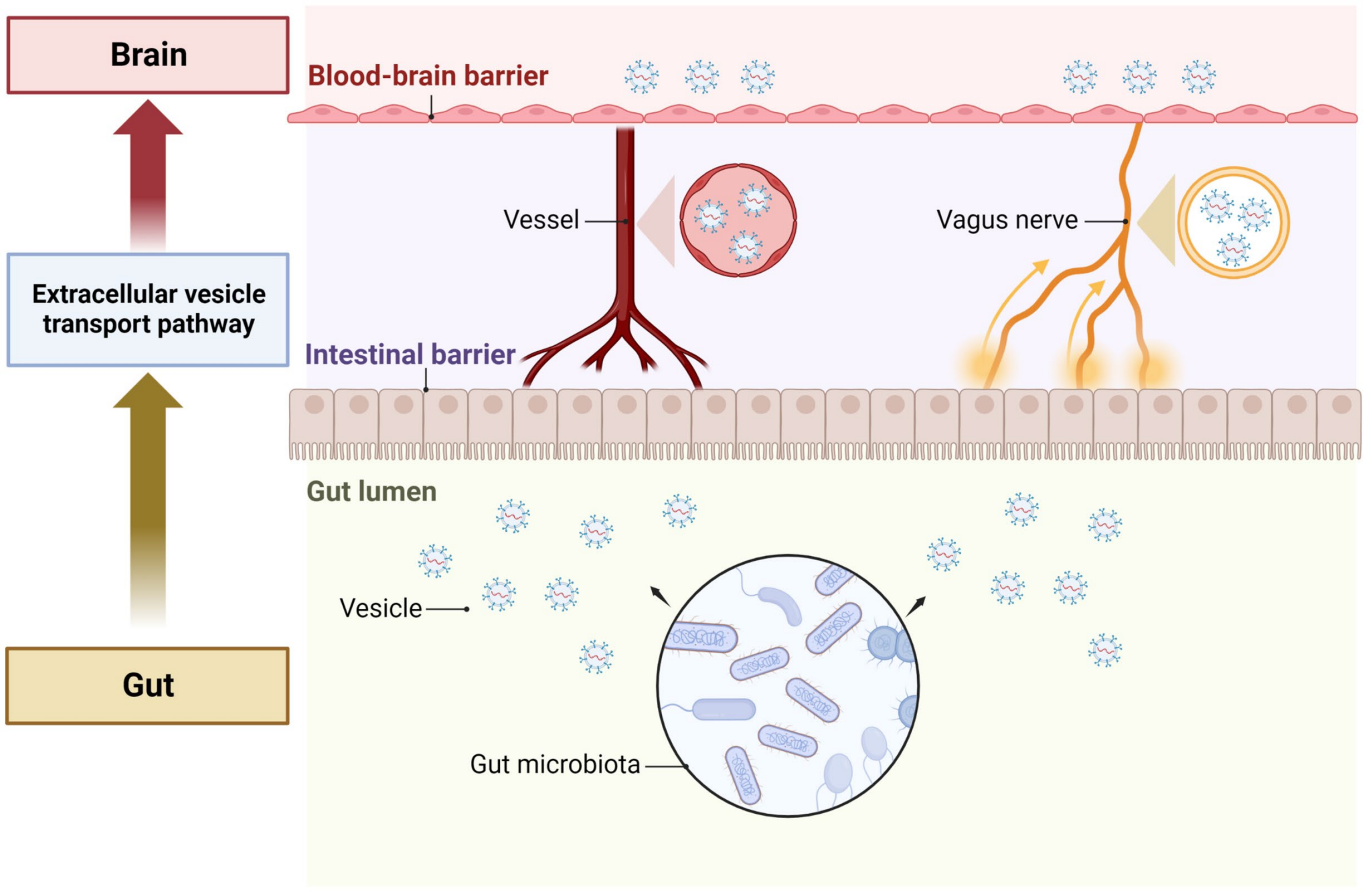
The role of GMEVs in mediating gut-brain communication. The gut microbiota release extracellular vesicles in the gut lumen. The vesicles pass through the cells of the intestinal epithelial layer lining the lumen and enter the blood vessels to be transported through the circulation. The vesicles then pass through the blood–brain barrier and enter the brain cells to exert their effects. In addition, vesicles can also be transported to the brain via the vagus nerves. GMEVs, gut microbiota-released extracellular vesicles.

Many studies have shown that gut microbiota plays an important role in central nervous system (CNS) regulation (Heiss and Olofsson, 2019). Intestinal microbiota can attract nerve signals between the brain and intestine through the interaction between the vagus nerve and the intestinal nervous system (Goehler et al., 2008; Kunze et al., 2009; Brookes et al., 2013; Perez-Burgos et al., 2013; Bonaz et al., 2018). The host’s endocrine response can transmit the signals of intestinal microorganisms to the brain through the circulation (Clarke et al., 2013; Martin et al., 2018). Intestinal microorganisms can regulate central and peripheral immune cells, leading to changes in stress and behavioral response. Metabolites released by intestinal microorganisms, such as neurotransmitters, can be circulated through the CNS (Lyte, 2011; Sarkar et al., 2016). Currently, the understanding of how signals transfer from the gut to the brain has become a priority area of scientific investigation. Based on the functions of GMEVs we discussed, the signal transduction from the gut to the CNS could be through GMEVs by following the nervous system or circulation pathways (Figure 2). A previous study showed that the EVs of *Paenalcaligenes hominins* from the intestine can cause vagus-dependent cognitive impairment (Lee et al., 2020). In addition, GMEVs from *Akkermansia muciniphila* were reported to induce the secretion of serotonin in the colon and hippocampus of mice and the Caco-2 cell line (Yaghoubfar et al., 2020). At present, these studies provide evidence to support that GMEVs could be a signal molecule that can control CNS.

## 6. Good GMEVs: probiotics-derived EVs and their potential therapeutic role in neurological disorders

**Figure 3:**
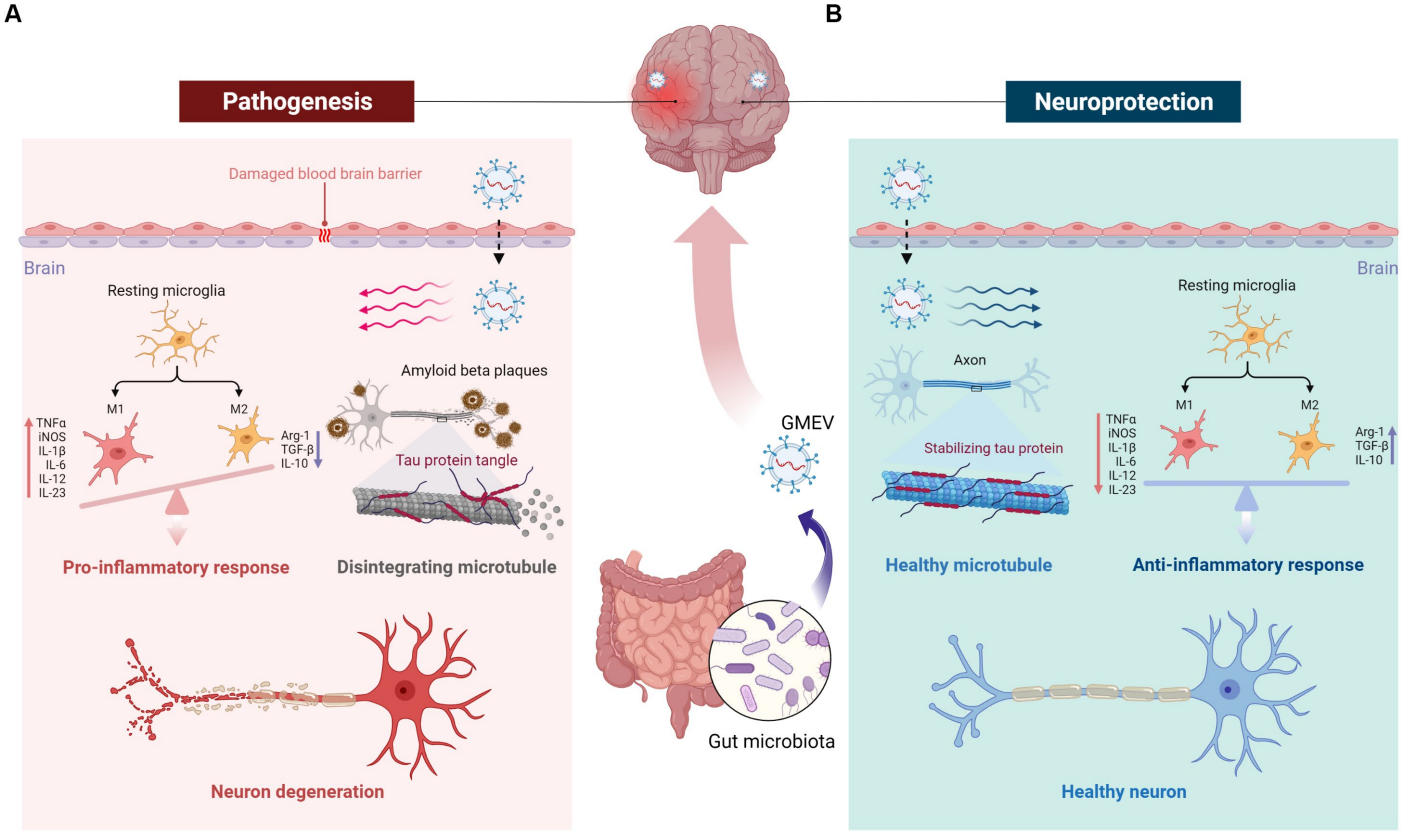
The double role of GMEVs in neurological disorders and the underlying mechanisms. GMEVs may aggravate or alleviate disease states indirectly via conferring harmful or beneficial effects. **(A)** Pathological GMEVs release their cargoes such as lipopolysaccharide that can cause disruption of the blood–brain barrier (BBB) and release of inflammatory cytokines, leading to activation of microglia and neuroinflammation. **(B)** The potential beneficial/therapeutic effects of GMEVs in neurological disorders could be via release protective lipids, small molecules, or microRNAs to further regulate downstream protective target proteins, thereby stabilizing the BBB, microglial balance, axon stability, and neuronal function. GMEVs, gut microbiota-released extracellular vesicles.

Although GMEVs cannot directly cause or treat diseases, they can indirectly regulate diseases via conferring harmful and beneficial effects (Figure 3). Gut probiotics often have beneficial neurological effects, some of which could be replicated by GMEVs (Al-Nedawi et al., 2015; Choi et al., 2019; West et al., 2020). EVs from probiotics may favorably modulate microglial function, exerting beneficial effects on the nervous system (Han et al., 2019; Wei et al., 2020). GMEVs, especially probiotic EVs, have emerged as a promising tool for therapeutic applications due to their nanosized structures, cell-free systems, drug loading capacity, low toxicity, and good biocompatibility.

### 6.1. Strokes

Ischemic stroke (IS) is a life-threatening cerebral vascular disease accounting for high disability and mortality worldwide. Emerging recent studies suggest a potential role of EVs in the treatment of IS Indeed, in recent years, various kinds of mammalian cell-derived EVs, such as those derived from mesenchymal stem cells, neural stem cells, type 2 microglia, and brain endothelial cells have been used to treat IS by altering the expression of microRNAs (miRs) in the organism (Xu et al., 2023). However, due to the prohibitive cost of mammalian cell culture, it is difficult to obtain a culture medium for EVs extraction on a large scale in the short term, which has limited the translation of EVs research to the clinic. Therefore, an alternative source of EVs with the obvious advantages of having quicker EVs extraction, at a low cost, and in large quantities would accelerate the development of novel therapeutic modalities for IS treatment. In this regard, a recent study has shown the efficacy of *Lactobacillus acidophilus*-derived EVs in protecting against ischemic brain injuries (Yang et al., 2022). *Lactobacillus acidophilus-*derived EVs can regulate c-Fos/TGF-β1 by targeting miR101a-3p to reduce neuronal apoptosis caused by IS. For now, the direct link between GMEVs and IS still needs to be further explored. Importantly, the characterization of the bioactive factors in these probiotics-derived EVs that mediate the ameliorating effects of IS will highlight newer avenues to develop novel therapeutic modalities.

### 6.2. Alzheimer’s disease

AD involves various changes in the brain, including the accumulation of abnormal protein deposits, neuroinflammation, synaptic dysfunction, and neuronal loss (Cervellati and Zuliani, 2023). These changes contribute to the progressive decline in cognitive function and memory loss characteristic of AD. Epidemiological studies show that intestinal bacterial infection will increase the prevalence of AD, but most intestinal bacteria do not directly invade the CNS (Hooi et al., 2017). The OMVs derived from gut microbiota are considered to play an important role in AD (Jaeger et al., 2009; Banks and Robinson, 2010). GMEVs may be an important driver of AD pathology and play an important role in AD’s pathogenesis, development, and defense. More importantly, GMEVs may also extend the pathological range of bacteria to the whole body, causing further cascade reactions and aggravating the injury. Although there is some evidence to suggest that OMV can promote inflammatory response and exacerbate adverse effects in AD, recent studies have shown that there are also some probiotics that can alleviate disease damage after AD, and their derived GMEVs may play an important role in this process. Nutritional supplements or medications containing probiotics can improve intestinal microbiota imbalance or neurological symptoms in AD patients. These effects may be caused by the recovery of gut microbiota and the regulatory changes in GMEVs in the gut-brain axis (Pluta et al., 2020). Although there is currently limited evidence that GMEVs directly regulate AD neurological disorders, the beneficial effects of probiotics on AD are suggestive of the potential role of probiotics-derived EVs in ameliorating AD-associated complications. Meanwhile, some studies have shown that in some diseases, probiotics can act through small molecule substances such as miRs and lipids encapsulated in GMEV (Díez-Sainz et al., 2022). For example, the EVs secreted by *A. actinomycetemcomitans* can reach the mouse brain and play a potential role in neuroinflammatory diseases through exogenous RNA cargo (Han et al., 2019).

### 6.3. Parkinson’s disease

PD is the second most common neurodegenerative disease, and the incidence rate continues to rise. Pathological signs of PD include nerve inclusion bodies in the form of Lewy body and cell loss in the neurite, substantia nigra, and other brain regions (Tolosa et al., 2021). Similar to AD, epidemiological studies also show that intestinal bacterial composition will affect the status of PD (Hooi et al., 2017). There is evidence even to implicate that PD may start in the gut and then spread to the brain through the vagus nerve (Holmqvist et al., 2014). In contrast, gut microbiota holds the promise of playing a beneficial role in PD and is likely associated with GMEV. *Lactobacillus plantarum* is an abundant family of probiotics, and multiple subtypes are neuroprotective in PD. *Lactobacillus plantarum* CCFM405 alleviates rotenone-induced PD mice via regulating gut microbiota and branched-chain amino acids biosynthesis (Chu et al., 2023). *Lactobacillus plantarum* DP189 reduces α-synuclein aggravation in MPTP-induced PD in mice via regulating oxidative damage, inflammation, and gut microbiota disorder (Wang et al., 2022). Although the above studies did not directly indicate the protective effect of *Lactobacillus plantarum-*derived EVs on PD, *Lactobacillus plantarum-*derived EVs have been proven to have clinical effects in other diseases, such as inhibiting wrinkle formation and pigmentation, and affecting inflammatory factors in macrophages generation (Aoki-Yoshida et al., 2017; Jo et al., 2022). Interactions between probiotics and host miRs in modulating host neuropathy are rare, as miRs are one of the common cargoes of GMEVs, and this study may implicate the role of GMEVs. Recently, GMEVs produced by intestinal microorganisms have been proven to be related to the inhibition of hypothalamic energy metabolism in major depressive disorder patients (Qi et al., 2020). For example, the GMEVs extracted from *Lactobacillus plantarum* have been shown to exhibit antidepressant-like activity in mice (Choi et al., 2019) supporting the potential use of GMEVs as a biological therapy for neurological disorder-induced depression.

Research on the beneficial effects of GMEVs on neurological disorders is still in its early stages, warranting detailed studies to define therapeutic potential and mechanisms of GMEVs in neurological disorders. While these findings suggest promising avenues for future investigations, the application of EV-based therapies in clinical settings is still a developing field.

## 7. Biomarker role of circulating GMEVs in neurological disorders

EVs are abundant in all body fluids, stable and small in size, and can easily cross physiological barriers, so they are considered to be a beneficial source of circulating biomarkers (van Niel et al., 2018). Since GMEVs are enriched by molecular content (Pathan et al., 2019), GMEVs are also considered biomarkers for many neurodegenerative diseases such as AD and PD (Yuyama et al., 2015; Kapogiannis et al., 2019; Pérez et al., 2019).

The contents of GMEVs exist in different forms of origin, either as components of the parental cell or as membrane-associated particles. During GMEVs biogenesis, different cargoes (i.e., mRNA, DNA, proteins, lipids, etc.) are loaded into vesicles; this can serve as a surrogate indicator of the parental cell to provide specific cell-of-origin biomarkers (Im et al., 2014; Keerthikumar et al., 2016). The study observed a correlation between GMEVs and the development of neurological syndromes like cerebral malaria, where carbohydrase 1 and S100A8 were identified as cargoes of cerebral malaria syndrome EV by proteomic analysis (Tiberti et al., 2016). They are specifically increased during pathogenesis, strengthening the notion of these molecules as biomarkers for malaria. Therefore, further research on GMEVs as biomarkers for more neurological disorders is promising.

## 8. Does the type of diet influence the type of EVs produced by gut bacteria?

The efficacy of GMEVs in conferring a beneficial or harmful effect on host health is species-specific and modulated by diet. Studies in mice have shown that a high-fat diet (HFD)-induced increase in the proteobacterium *Pseudomonas panaceas*–derived EVs is strongly associated with the progression of metabolic disorders such as obesity, diabetes, and hypertension, the known risk factors of stroke (Choi et al., 2011). In contrast, *Akkermansia muciniphila* EVs are linked with alleviating HFD-induced metabolic dysfunctions (Yaghoubfar et al., 2020). Therefore, detailed mechanistic studies utilizing experimental models of stroke are warranted to select the probiotic bacterial species having beneficial effects on neuron/brain function and to unravel the critical role of their EVs in alleviating the risk factors for IS and/or the unfavorable post-stroke outcomes.

Importantly, glial cells in CNS have been proven to participate in weight homeostasis and obesity, while a HFD can usually induce obesity and metabolic syndrome, so diet has the potential to regulate the neuroinflammation (Horvath et al., 2010; García-Cáceres et al., 2012; Valdearcos et al., 2017). There is a close relationship between diet and intestinal flora, but little is known about the regulation of GMEVs by diet. Sundaram et al. developed a kind of garlic exosome-like nanoparticles (GaELNs) and administered them orally to mice on a high-calorie diet (Sundaram et al., 2022). This study’s results showed that GaELNs can preferentially ingest microglia and inhibit brain inflammation in HFD mice. GaELN phosphatidic acid forms a complex through the interaction with microglial cell brain acid soluble protein 1 and inhibits the expression of c-Myc-mediated STING, resulting in the reduction of the expression of a series of inflammatory cytokines, thus promoting neuronal differentiation and inhibiting mitochondria-mediated neuronal cell death. Although the role of GMEVs in this study is unknown, it is also an indisputable fact that EVs can regulate the neuroinflammation of mice under HFD through oral action on the intestine, which may lay a foundation for us to study the regulation of GMEVs by diet in the next step to regulate nervous system diseases.

## 9. Engineering bacteria to produce beneficial GMEVs

**Figure 4:**
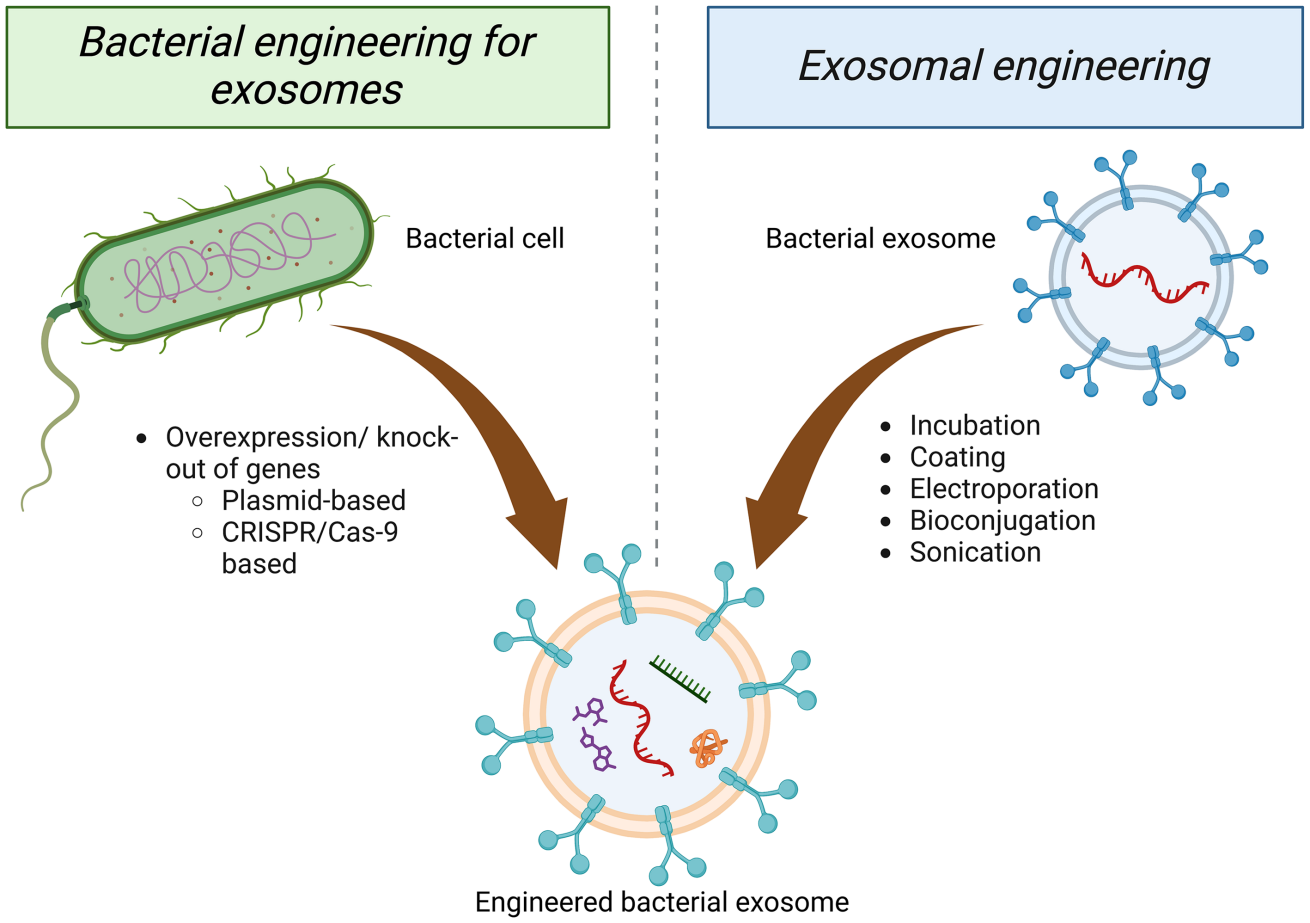
Approaches of engineering GMEVs. Engineering of GMEVs to confer superior functionality/beneficial effects can be achieved mainly via two approaches, either through the parental bacterial cell or isolated GMEVs. Engineering of the parental bacteria can be conducted via targeting bacterial cells for plasmid-based or CRISPR/Cas-9-based overexpression/knock-out of specific genes. The engineering of GMEVs can be achieved via various techniques such as—incubation, coating, electroporation, bioconjugation, sonication, etc. with specific genes, chemical drugs, genetic materials, etc. GMEVs, gut microbiota-released extracellular vesicles.

As biologically derived entities, GMEVs can be modified to achieve enhanced desirable attributes through molecular biology and bioengineering technology. Fundamentally, GMEVs may be modified via two approaches; engineering parental strain to create superior therapeutic EVs and engineering EVs after isolation to improve their functionality (Figure 4). *Escherichia coli* provides a broad and flexible platform for the development of bioengineered OMVs. The system contains a variety of target proteins that can be fused with OMVs-related toxin cytolysin A, including β-lactamase, organophosphate hydrolase, green fluorescent protein, and Fc antibody fragments. This system enables the display of chimeras on the OMVs’ surface, preserving target proteins and OMVs’ function (Kim et al., 2008).

In addition, GMEVs can be loaded with therapeutics, and their cohesins, sulfatases, and proteases, facilitating interactions with and enter host epithelial cells, making them potentially effective drug delivery vehicles (Shen et al., 2012; Elhenawy et al., 2014; Hickey et al., 2015; Stentz et al., 2015). Importantly, drug delivery to the brain is usually limited by the BBB, and OMVs have great potential because of their ability to cross the BBB (Dong, 2018; Wei et al., 2020). Chen et al. applied a mixture of nano-gold and GMEVs to enhance the radiotherapy and immunotherapy effect against glioblastoma and prolong the survival period of mice (Chen et al., 2021). In addition, OMVs can be taken up by surrounding neutrophils and signal to microglia, and thus may be able to deliver drugs to microglia (Li et al., 2020). OMVs have been loaded with drugs or siRNA for targeted therapy in cancer treatment, and we expect to be able to target OMVs to the brain for the treatment (Shi et al., 2016; Kim et al., 2017).

Gut microbiota dysbiosis may contribute to neurological diseases, and the targeted application of GMEVs as therapeutic vaccines may be beneficial. In this case, the vaccine does not eliminate the pathogen itself but instead inhibits the active molecules released by the pathogen, leading to deleterious remodeling of the microbiota and subsequent disease onset (Pirolli et al., 2021). For example, an EcN OMVs vaccine was used to generate a broadly protective immune response to conserved microbial sugars that outperformed conventional vaccines, which have historically been costly and laborious to produce (Stevenson et al., 2018).

## 10. Conclusions and future perspectives

GMEVs have long been viewed as a double-edged sword for the gut-brain axis. The downside is that GMEVs can act as a causative factor to activate harmful neuroinflammation, thereby inducing the pathology of some neurodegenerative diseases, especially AD. On the plus side, the carrier role of GMEVs and their ability to cross biological barriers can amplify their specific immunomodulatory potential, which is expected to induce protective immunity and target biological barriers (such as the BBB) in a variety of diseases for drug delivery. In particular, probiotic GMEVs or OMVs vaccines can induce broad protective effects to improve microbiota dysbiosis, enhance the body’s defense mechanisms against pathogenic gut microbes, and even directly target brain therapy. At present, the value of GMEVs as a therapeutic carrier and therapeutic target for neurodegenerative diseases has been explored to some extent. But the relationship of GMEVs to genetic risk factors for neurodegeneration remains to be resolved. Furthermore, how GMEVs play a role in other neurological disorders, including IS, still needs to be explored. Brain injury has been shown to lead to intestinal chronic changes in the microbiome, which in turn may further propagate neurological damage and suggest a potential role for GMEVs in brain injury, especially IS, traumatic brain injury (Peh et al., 2022). The transportation and transmission efficiency of GMEVs-mediated signaling molecules are also lacking for further research, and how to use bioengineering techniques to decorate GMEVs to play their beneficial role also needs to be explored.

In summary, GMEVs, due to their similar composition to the gut microbiota, can replicate the deleterious and beneficial effects of pathogenic and probiotic bacteria and can directly affect neurological disorders. Moreover, due to their special carrier role, they have the potential to spread over long distances in the body, cross the BBB and induce neuroimmune responses that alter neurological function. Therefore, how to limit its bad effects, further exert its beneficial effects, and explore its role in more types of neurological diseases are the avenues for further extensive investigation.

## Author contributions

BS and HS searched the literature and drafted the manuscript. AB and JB critically revised the manuscript. All authors contributed to the article and approved the submitted version.

## Funding

This work was supported by the National Institute of Neurological Disorders and Stroke (1R01NS102720), Chronic Disease Research Program (WV-INBRE grant, P20GM103434) and West Virginia Clinical and Translational Science Institute (WV-CTSI) grant (2U54GM104942) to JB, National Institute of Allergy and Infectious Disease (AI130790-03), and National Institute of General Medical Sciences (P20 GM 121299-04) to AB.

## Conflict of interest

The authors declare that the research was conducted in the absence of any commercial or financial relationships that could be construed as a potential conflict of interest.

## Publisher’s note

All claims expressed in this article are solely those of the authors and do not necessarily represent those of their affiliated organizations, or those of the publisher, the editors and the reviewers. Any product that may be evaluated in this article, or claim that may be made by its manufacturer, is not guaranteed or endorsed by the publisher.
